# Supplementary material for: Early life environment moderates association of body composition and internalizing problems in adolescence
Source: Commun Psychol. 2025 Nov 20;3:163. doi: 10.1038/s44271-025-00336-0 (PMC12634448; doi:10.1038/s44271-025-00336-0)
Supplement: Supplementary file 2 — Supplemental Materials [file 44271_2025_336_MOESM2_ESM.pdf]

Supplementary Materials S1: Data Preprocessing Procedures

Data Preprocessing: Covariates of Interest (Body-Mass-Index and Waist-to-Height Ratio)

BMI and WHtR were derived from lab-based measurement of height (ABCD variable name: anthroheightcalc), weight (anthroweightcalc), and waist circumference (anthro_waist_cm). Height and weight were acquired in duplicate or triplicate and averaged as follows. If three measurements were obtained, the two closest measurements were averaged. If the third measurement fell equally between the first two measurements, all three were averaged. Waist circumference was measured by study staff at the position of the iliac crest using measuring tape at 0.1cm increments. Height, weight, and waist measurements were available for 99%, 93%, and 93% of visits, respectively. BMI was based on measured height and weight in imperial units (703*weight/height^2). Extreme outliers were removed from analysis using the following criteria based on visual inspection: weight less than 45 lbs, height less than 45 inches or more than 72 inches, waist circumference less than 19 inches, BMI less than 10 or greater than 45, and WHtR less than 0.3 or greater than 0.9. Collectively, these criteria excluded roughly 1.1% of available data.

Data Preprocessing: Outcomes (Internalizing Behaviors)

Internalizing scores quantify anxious, depressive, and somatic complaint behaviors, and were based on the ABCD-provided parent-based Child Behavior Checklist (CBCL) raw internalizing behavior score (mh_p_cbcl/cbcl_scr_syn_internal_r). The CBCL is routinely used for research and clinical purposes with intraclass correlations above .90 for interparent agreement, 1-week test-retest reliability, and inter-interviewer reliability(1, 2). Raw scores (rather than standardized scores) were chosen based on having a sufficient sample size to simultaneously model (potentially) non-linear sample-based age, sex, puberty, and demographic effects. Inverse hyperbolic sine and rank inverse norm transformations were used to bring the distributions toward normality and considered in supplementary analyses, along with ABCD-provided t-scores. Within-person WHtR slopes were derived based on a linear mixed effects model and reflects the fit/slope of repeated WHtR and internalizing behavior measures within an individual. Internalizing behavior scores were available for 89.2% of visits.

Data Preprocessing: Moderators (Adverse Childhood Experiences and Protective Environments)

Negative and positive influences (ABCD-ACEs and protective environment scores and items) were considered as effect modifiers at the between- and within-person levels. ABCD-ACEs were scored based on prior literature conducted using ABCD data.(3) This "ABCD-ACEs" score includes domains conceptually aligned with the original ACEs categories. However, the ABCD-ACEs score expands this framework to include additional adversities relevant to the ABCD cohort, such as exposure to discrimination, bullying, natural disasters, and significant accidents. These additions reflect a broader but still conceptually grounded operationalization of childhood adversity using item-level data. In brief, ABCD-ACEs were coded from item-level responses collected at the baseline and 1-year assessments. A cumulative risk score based on a mixture of parent and child report of having experienced an event from the following categories was used: emotional/physical/sexual abuse; household substance use; household mental illness; parental separation; family involvement with legal system; emotional/physical neglect; extreme financial adversity; discrimination; bullying; domestic violence; grief; community violence; natural disaster; witnessing war zone trauma; witnessing an act of terrorism; car accident; or other significant accident requiring medical attention. Raw cumulative risk scores ranged from 0-19 (with a maximum possible of 21). ABCD-ACEs were available for all participants with anthropometric data.

Protective environments were based on prior literature conducted using ABCD data.(4) Representative measures were pulled from four consortium-recommended domains focused on family cohesion (ce_p_fes/fes_p_ss_cohesion_sum_pr), peer involvement (ce_y_pbp/pbp_ss_prosocial_peers), school risk and protective factors (ce_y_srpf/srpf_y_ss_ses), and social cohesion at the neighborhood level (ce_p_comc/comc_ss_cohesion_p). Family cohesion subscales were not available for the youth report

version of the family environment scale. These measures were modeled at increasingly granular levels as follows: first in aggregate based on a factor analysis score, next at the domain level (family, peers, school, community), and finally at the single item level to inform on specific intervention points. These instruments were collected at baseline and two-year follow-up visits and collapsed into a single score for each individual.

Data Preprocessing: Covariates of Non-Interest (Potentially Confounding Factors)

Covariates of non-interest were chosen to prevent hidden confounding and account for developmental variance. These measures were selected based on published literature examining socioeconomic status in the context of obesity and cognition(5), and included age at interview, biological sex at birth, puberty status (visit-level specific, ABCD provided categorical measure, pds_p_ss_female_category_2 and pds_p_ss_male_category_2), self-reported race (collapsed into Black, White, Other based on prior ABCD practices)(5) and ethnicity, area deprivation index (baseline reported), household income, and maximum parental education (values below high-school level were collapsed into a single category). Observations where participants chose not to respond were removed for analysis as shown in Figure 2.

Supplementary Materials S2: Mixed Effects Generalized Additive Model Selection

Model selection was performed to further understand the relationship of WHtR and BMI with internalizing behaviors across adolescence. This process began based on the ABCD recommendation of using Mixed Effects Generalized Additive Models and focused on identifying significant non-linear patterns between the predictors and outcomes. To do this, we chose a model decomposing first order (linear) terms from spline basis functions capturing second order polynomials and higher (Supplementary Table S1). Note that WHtR had a surviving linear component while BMI did not, and that only BMI had a significant non-linear relationship with internalizing behaviors (Supplementary Figure S1). While the interpretation of this is that representing WHtR as a spline-based function is not the most parsimonious choice, we chose to keep its use when comparing its performance against a spline-based representation of BMI, and as a linear term when not. Further, while a higher-order term for household income was significant at the $p<0.05$ threshold in this initial model, it did not consistently exhibit non-linear behavior (*i.e.,* it was model dependent) and therefore was modeled linearly throughout.

Further analysis considered an optimal model that either included BMI or WHtR in isolation, or together. To do this, we began with a parsimonious model considering only covariates of non-interest to identify a baseline measure of explanatory performance (Supplementary Table S2). Next, we added BMI in isolation (Supplementary Table S3), followed by WHtR in isolation (Supplementary Table S4). Finally, an omnibus model considering both in the same model was considered (Supplementary Table S5). Model performance (Bayesian Information Criterion) and additional explained variance over the parsimonious model were considered in aggregate (Table 2) and cross-sectionally (Supplementary Figures S2 and S3). Collectively, these analyses suggested that while non-linear aspects of BMI capture a small degree of additional variance, the majority of variance is most parsimoniously explained by a linear relationship with WHtR. Thus, this model was considered as the optimal model for reporting here and carrying forward to further conduct between- and within-person analyses.

Finally, because the residuals from the optimal model were not normally distributed, we performed sensitivity analyses to confirm that alternative forms of the outcome (internalizing behaviors) and error structures (Gaussian vs. Poisson) did not change the primary conclusions made here. For the alternative forms of the outcome, we considered three different transformations aimed at forcing a more normally distributed outcome (and therefore residual): ABCD-provided t-scores, a van der Waerden (rank inverse) transformation, and a asinh transformation. WHtR remained significantly associated ($p<10^{-15}$) with internalizing behaviors under all transformations and error models. To compare effect sizes across different transformations we report F-scores ($F_{raw\text{-}score,manuscript}=58.4$; $F_{t\text{-}score}=44.1$; $F_{vdW\text{-}score}=42.4$; $F_{asinh\text{-}score}=36.1$;). Collectively, these support that WHtR is robustly associated with internalizing behaviors despite model choice and error structure.

Independent Variable	Estimate	95% CI	t-value(df)
(Intercept)	4.266	[1.82 6.72]	3.414***
Age at Assessment	-0.003	[-0.01 0.00]	-1.019
Biological Sex at Birth (Male)	-0.144	[-0.27 -0.01]	-2.196*
Puberty	0.208	[0.14 0.27]	6.199***
Area Deprivation Index	0.008	[0.00 0.013]	2.660**
Parental Education (ref. Bachelor's Degree)			
≤ High School Graduate	-0.832	[-1.17 -0.49]	-4.803***
High School Graduate	-1.087	[-1.37 -0.81]	-7.618***
GED or Equivalent	-0.841	[-1.23 -0.46]	-4.300***
Some College	0.077	[-0.13 0.28]	0.738
Associates Degree Occupational	0.009	[-0.23 0.24]	0.071
Associates Degree Academic	0.327	[0.07 0.58]	2.498*
Master's Degree	-0.028	[-0.18 0.13]	-0.358
M.D. or Equivalent	-0.259	[-0.530.016]	-1.846
Ph.D. or Equivalent	-0.015	[-0.27 0.24]	-0.115
Household Income	-0.238	[-0.34 -0.13]	-4.450***
Self-report Ethnicity (Hispanic)	-0.035	[-0.19 0.12]	-0.437
Self-report Race (ref. Black)			
Other/Unknown	0.936	[0.67 1.20]	7.004***
White	1.736	[1.55 1.92]	18.722***
Body-Mass-Index	-0.049	[-0.15 0.055]	-0.926
Waist-to-Height Ratio	3.378	[1.96 4.79]	4.682***
**Smooth Term**	**edf**	**Ref.df**	**F**
s(Body-Mass-Index)	3.964	8	0.808***
s(Waist-to-Height Ratio)	0.103	8	0.012
s(Age at Assessment)	0.575	8	0.081
s(Area Deprivation Index)	0.698	8	0.036
s(Household Income)	1.886	8	0.287*

Supplementary Table S1. Internalizing Behaviors: Model Statistics for Selecting Non-linear Terms. A mixed effects generalized additive model was used to identify significant linear and non-linear (2nd order splines or higher) terms. Significance codes: * p<0.05, ** p<0.01, *** p<0.001. N=31,418 observations across 10,446 unique individuals were considered. The model degree of freedom for parametric terms (df) is 31,393.

Independent Variable	Estimate	95% CI	t-value(df)
(Intercept)	4.374	[3.75 4.99]	13.831***
Age at Assessment	-0.001	[-0.00 0.00]	-0.355
Biological Sex at Birth (Male)	-0.158	[-0.29 -0.03]	-2.417*
Puberty	0.210	[0.15 0.27]	6.434***
Area Deprivation Index	0.010	[0.01 0.01]	6.644***
Parental Education (ref. Bachelor's Degree)			
≤ High School Graduate	-0.741	[-1.08 -0.40]	-4.299***
High School Graduate	-0.940	[-1.22 -0.66]	-6.606***
GED or Equivalent	-0.724	[-1.11 -0.34]	-3.707***
Some College	0.201	[-0.00 0.40]	1.934
Associates Degree Occupational	0.122	[-0.11 0.36]	1.020
Associates Degree Academic	0.443	[0.19 0.70]	3.387***
Master's Degree	-0.073	[-0.23 0.08]	-0.933
M.D. or Equivalent	-0.347	[-0.62 -0.07]	-2.503*
Ph.D. or Equivalent	-0.117	[-0.37 0.14]	-0.892
Household Income	-0.233	[-0.27 -0.20]	-13.078***
Self-report Ethnicity (Hispanic)	0.092	[-0.06 0.25]	1.176
Self-report Race (ref. Black)			
Other/Unknown	0.851	[0.59 1.11]	6.409***
White	1.641	[1.46 1.82]	17.973***

Supplementary Table S2. Internalizing Behaviors: Model Statistics Using Linear Terms. Summary statistics for the ME-GAM model including covariates of non-interest used to model the effects of BMI and WHtR used throughout the manuscript. Significance codes: * $p<0.05$, ** $p<0.01$, *** $p<0.001$. N=31,418 observations across 10,446 unique individuals were considered. The model degree of freedom for parametric terms (df) is 31,400.

Independent Variable	Estimate	95% CI	t-value(df)
(Intercept)	5.140	[4.51 5.77]	15.988***
Age at Assessment	-0.006	[-0.01 -0.00]	-3.268**
Biological Sex at Birth (Male)	-0.139	[-0.27 -0.01]	-2.125*
Puberty	0.198	[0.13 0.26]	5.947***
Area Deprivation Index	0.009	[0.01 0.01]	5.656***
Parental Education (ref. Bachelor's Degree)			
≤ High School Graduate	-0.870	[-1.21 -0.53]	-5.057***
High School Graduate	-1.087	[-1.37-0.81]	-7.632***
GED or Equivalent	-0.835	[-1.22 -0.45]	-4.281***
Some College	0.121	[-0.08 0.32]	1.170
Associates Degree Occupational	0.055	[-0.18 0.29]	0.458
Associates Degree Academic	0.374	[0.12 0.63]	2.865**
Master's Degree	-0.053	[-0.21 0.10]	-0.671
M.D. or Equivalent	-0.323	[-0.60 -0.05]	-2.329*
Ph.D. or Equivalent	-0.071	[-0.33 0.19]	-0.544
Household Income	-0.230	[-0.27 -0.19]	-12.929***
Self-report Ethnicity (Hispanic)	0.007	[-0.15 0.16]	0.088
Self-report Race (ref. Black)			
Other/Unknown	0.979	[0.72 1.24]	7.376***
White	1.770	[1.59 1.95]	19.339***
**Smooth Term**	**edf**	**Ref.df**	**F**
s(Body-Mass-Index)	5.154	5.154	38.27***

Supplementary Table S3. Internalizing Behaviors: Model Statistics for Mixed Effects Generalized Additive Model Including Body-Mass-Index. Summary statistics for the ME-GAM model including BMI only. Significance codes: * p<0.05, ** p<0.01, *** p<0.001. N=31,418 observations across 10,446 unique individuals were considered. The model degree of freedom for parametric terms (df) is 31,395.

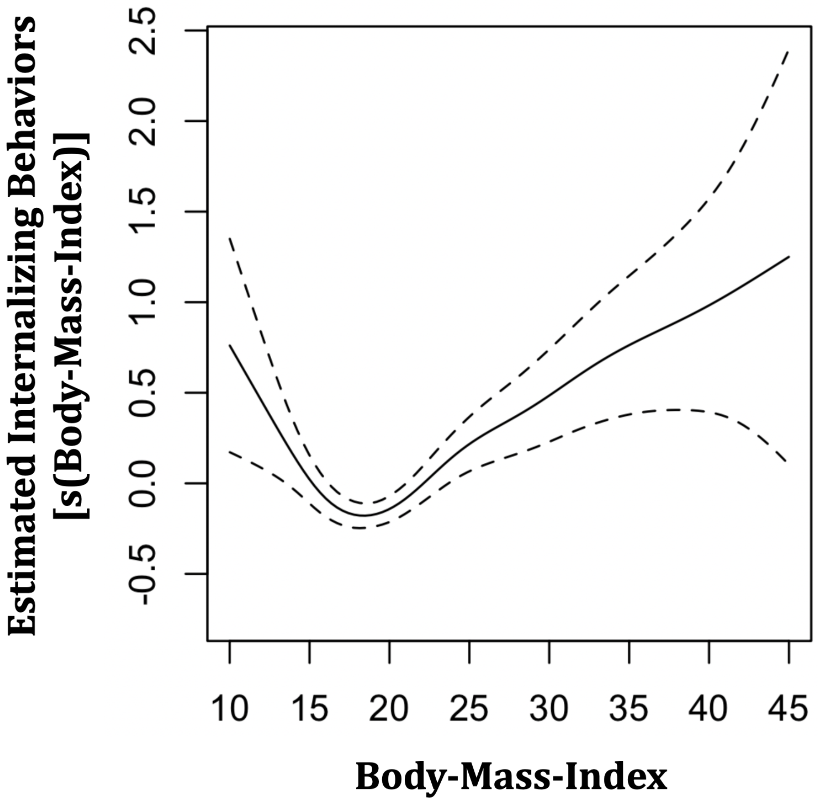

Supplementary Figure S1. Smoothed Non-linear Body-Mass-Index (BMI) Associations. BMI was non-linearly (roughly parabolic) associated with internalizing behaviors. N=31,418 observations across 10,446 unique individuals were considered.

Independent Variable	Estimate	95% CI	t-value(df)
(Intercept)	4.640	[4.02 5.26]	14.670***
Age at Assessment	-0.002	[-0.01 0.00]	-1.091
Biological Sex at Birth (Male)	-0.160	[-0.29 - 0.03]	-2.444*
Puberty	0.210	[0.15 0.28]	6.431***
Area Deprivation Index	0.009	[0.01 0.01]	5.665***
Parental Education (ref. Bachelor's Degree)			
≤ High School Graduate	-0.861	[-1.20 - 0.52]	-5.002***
High School Graduate	-1.073	[-1.35 - 0.79]	-7.535***
GED or Equivalent	-0.826	[-1.21 - 0.44]	-4.230***
Some College	0.129	[-0.07 0.33]	1.241
Associates Degree Occupational	0.055	[-0.18 0.29]	0.456
Associates Degree Academic	0.375	[0.12 0.63]	2.874**
Master's Degree	-0.054	[-0.21 0.10]	-0.683
M.D. or Equivalent	-0.332	[-0.60 - 0.06]	-2.398*
Ph.D. or Equivalent	-0.068	[-0.320.19]	-0.519
Household Income	-0.227	[-0.26 - 0.19]	-12.734***
Self-report Ethnicity (Hispanic)	-0.010	[-0.16 0.145]	-0.121
Self-report Race (ref. Black)			
Other/Unknown	0.900	[0.64 1.16]	6.789***
White	1.688	[1.51 1.87]	18.494***
**Smooth Term**	**edf**	**Ref.df**	**F**
s(Waist-to-Height Ratio)	3.10	3.10	58.44***

Supplementary Table S4. Internalizing Behaviors: Summary of Mixed Effects Generalized Additive Model Including Waist-to-Height Ratio. Summary statistics for the ME-GAM model including WHtR. Significance codes: * p<0.05, ** p<0.01, *** p<0.001. N=31,418 observations across 10,446 unique individuals were considered. The model degree of freedom for parametric terms (df) is 31,397.

Independent Variable	Estimate	95% CI	t-value(df)
(Intercept)	4.819	[4.17 5.46]	14.654***
Age at Assessment	-0.003	[-0.01 0.00]	-1.838
Biological Sex at Birth (Male)	-0.144	[-0.27 -0.01]	-2.189*
Puberty	0.211	[0.15 0.28]	6.295***
Area Deprivation Index	0.008	[0.00 0.01]	5.560***
Parental Education (ref. Bachelor's Degree)			
≤ High School Graduate	-0.879	[-1.22 -0.54]	-5.109***
High School Graduate	-1.096	[-1.37 -0.82]	-7.695***
GED or Equivalent	-0.843	[-1.23 -0.46]	-4.319***
Some College	0.115	[-0.09 0.32]	1.105
Associates Degree Occupational	0.046	[-0.19 0.28]	0.382
Associates Degree Academic	0.363	[0.11 0.62]	2.779**
Master's Degree	-0.049	[-0.20 0.10]	-0.623
M.D. or Equivalent	-0.324	[-0.59 -0.05]	-2.342*
Ph.D. or Equivalent	-0.064	[-0.32 0.19]	-0.488
Household Income	-0.228	[-0.26 -0.19]	-12.815***
Self-report Ethnicity (Hispanic)	-0.011	[-0.17 0.14]	-0.145
Self-report Race (ref. Black)			
Other/Unknown	0.942	[0.68 1.20]	7.094***
White	1.733	[1.55 1.91]	18.890***
**Smooth Term**	**edf**	**Ref.df**	**F**
s(Body-Mass-Index)	4.988	4.988	8.858***
s(Waist-to-Height Ratio)	1.004	1.004	26.512***

Supplementary Table S5. Internalizing Behaviors: Model Statistics for Mixed Effects Generalized Additive Model Including Body-Mass-Index (BMI) and Waist-to-Height Ratio (WHtR). Summary statistics for the ME-GAM model including both BMI and WHtR. Note that while BMI and WHtR do demonstrate independent variance associated with internalizing behaviors, WHtR does so in a more linear (low estimated degree of freedom [edf] value) and efficient (high F-score) manner. Significance codes: * p<0.05, ** p<0.01, *** p<0.001. N=31,418 observations across 10,446 unique individuals were considered. The model degree of freedom for parametric terms (df) is 31,394.

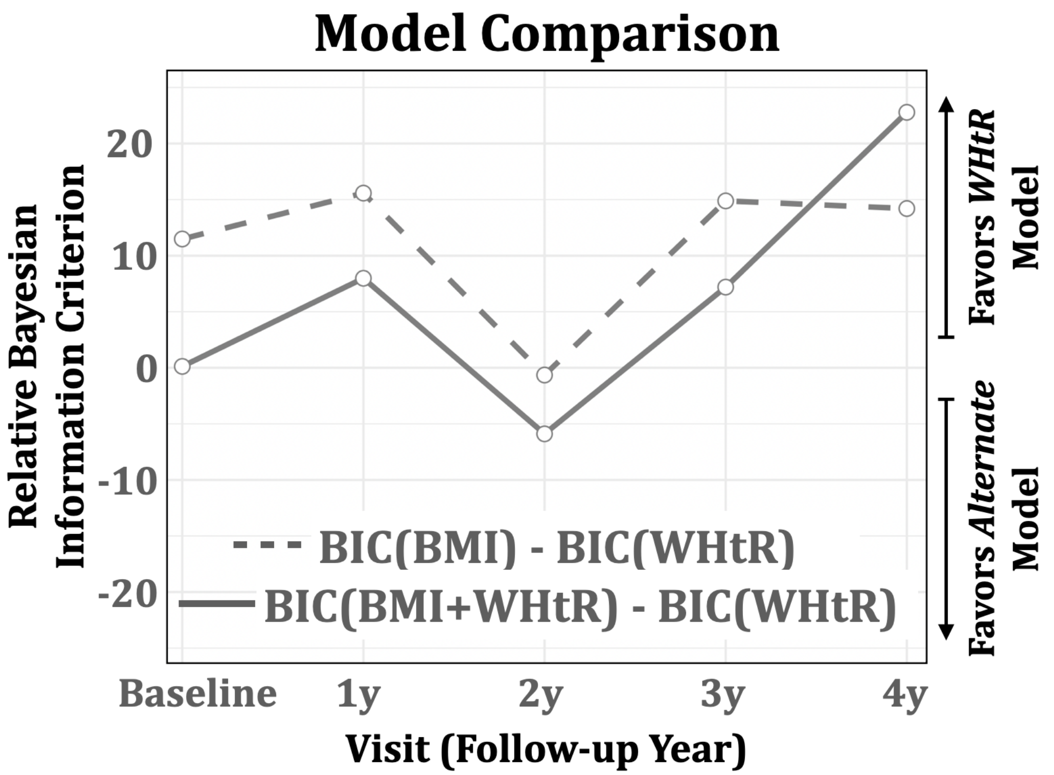

Supplementary Figure S2. Cross-Sectional Model Comparison: BIC. Models were compared by their Bayesian Information Criterion (BIC) score. With the exception of the 2y follow-up visit, WHtR was the optimal model when comparing against BMI alone or together with BMI (BMI+WHtR). Observations considered at each visit were n_{base}=9,898; n_{1y}=9,335; n_{2y}=7,666; n_{3y}=1,630; n_{4y}=2,839.

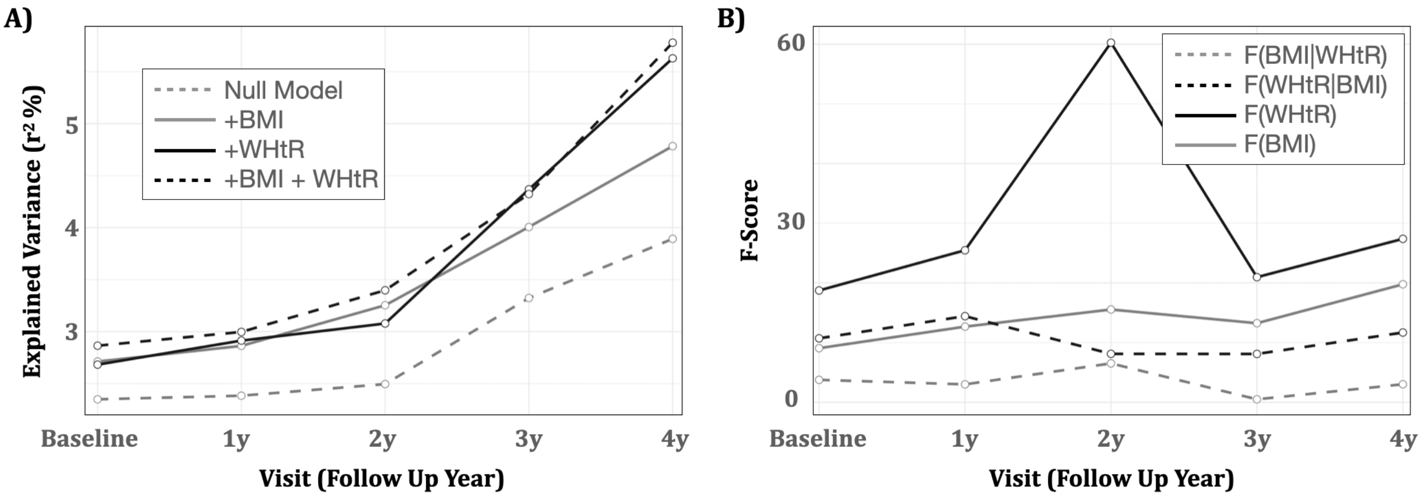

Supplementary Figure S3. Cross-Sectional Model Comparison: Explained Variance and F-score. Models were compared by their explained variance (panel A), and non-linear GAMM F-score (panel B). In brief, the model specified using WHtR alone demonstrated comparable or better prediction (explained variance) and effect size (F-score, a measure of efficiency of predictor relative to non-linear degrees of freedom), relative to BMI alone. In addition, model performance using WHtR alone demonstrated comparable prediction when compared to a model including both BMI and WHtR. Collectively this then supports WHtR alone as a more parsimonious and effective model than either BMI alone or WHtR in combination with BMI. F-statistics shown are from nested linear models, testing each anthropometric measure's independent effect when the other is included as a covariate. F(BMI | WHtR): F-statistic for BMI in a model adjusting for waist-to-height ratio. F(WHtR | BMI): F-statistic for waist-to-height ratio in a model adjusting for BMI. Larger F values indicate a stronger unique association with the outcome. Observations considered at each visit were n_{base}=9,898; n_{1y}=9,335; n_{2y}=7,666; n_{3y}=1,630; n_{4y}=2,839.

Supplementary Materials S3: Sensitivity Analyses: Considering Independent Raters of Internalizing Behaviors

Primary analyses conducted here relied on caregiver/adult-report of internalizing behaviors. While we believe this to be the most appropriate choice for assessment and the current ABCD/community standard, we also considered youth(self)- and teacher-report based on the Brief Problem Behavior survey (*bpm_y_scr_internal_r* and *bpm_t_scr_internal_r*). In brief, self-report may be more proximal to internal states of mind, whereas teacher-report provides a more objective assessment in a structured setting.

We first considered to what degree these ratings correlated with one another using a standard Pearson Correlation coefficient (Supplementary Figure S4). Second, we reran analyses using different raters for the internalizing behavior outcomes. In brief, ratings were highly rater dependent and did not share a large degree of variance. Despite this, WHtR was robustly associated with outcomes using both self- and teacher-report (Supplementary Table S6), suggesting that this association is rater-independent. Because the CBCL-based score is a longer instrument capturing internalizing behaviors at a finer scale and with more complete data, we continued to use the CBCL-based assessment throughput.

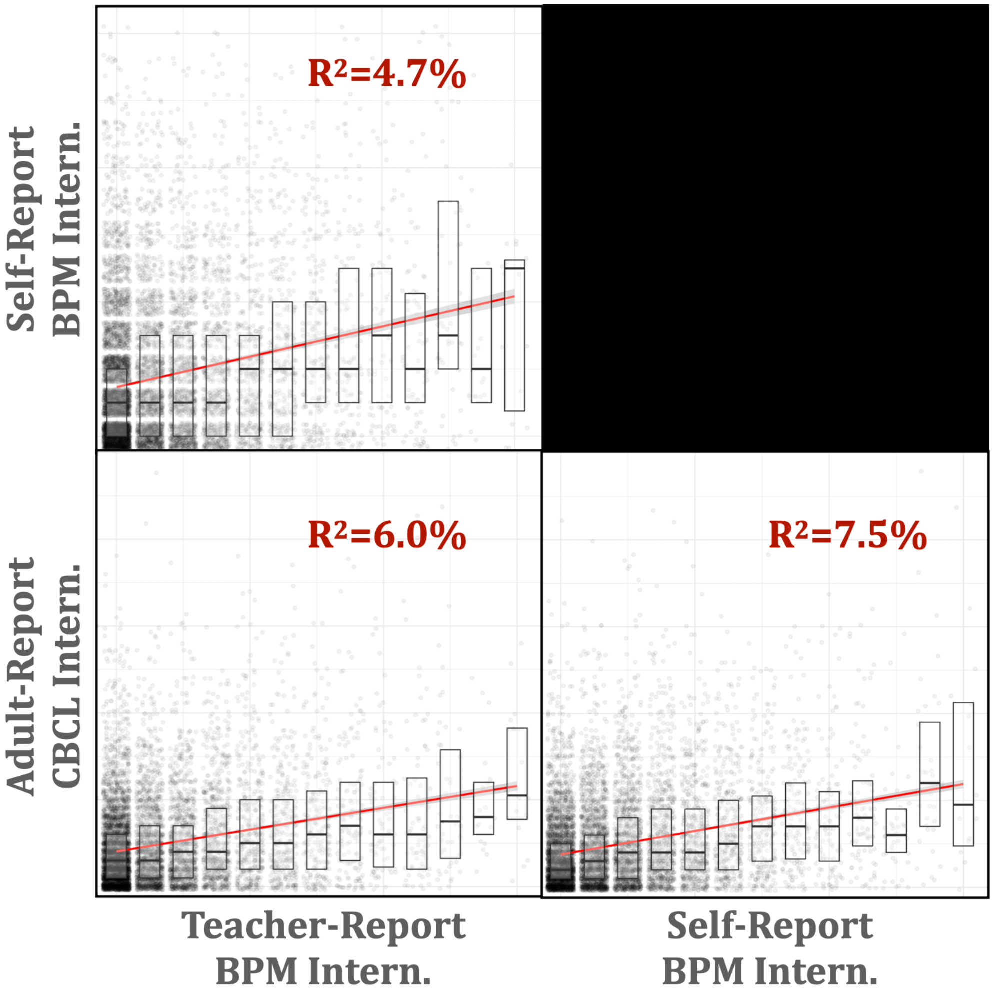

Supplementary Figure S4. Inter-rater Correlation Matrix: Internalizing Behaviors. Correlations between adult-, self-, and teacher-reported internalizing behaviors are shown.

Rater/Survey	$n_{participants}$	$n_{observations}$	F-score	p-value
**Adult/CBCL**	10,446	31,418	58.4	$<10^{-15}$
**Self/BPM**	9,752	20,327	40.2	$<10^{-15}$
**Teacher/BPM**	8,119	14,270	18.2	$<10^{-15}$

Supplementary Table S6. Associations Between Internalizing Behaviors and Waist-to-Height Ratio (WHtR): Rater Independence. Summary statistics for the ME-GAM association between internalizing behaviors and WHtR are provided. F-statistics are provided for effect size comparison.

Supplementary Materials S4: Testing for Age- and Sex-Specific Associations

Based on the logic that there are potential developmental and sex differences in puberty, body composition, and internalizing behaviors, we tested for age- and sex-specific effects in the context of the association between WHtR and internalizing behaviors. This was done in two ways. First, because interactions with spline-based GAMs are complicated, and because WHtR takes on a predominantly linear association with internalizing behaviors, we tested for age by WHtR, and sex by WHtR interactions in a standard linear mixed effects regression model. Significance testing was based on ANOVA χ^2 statistics using nested models. Second, because our primary model used here (for comparison against BMI) was a GAM, we also stratified by sex and performed a qualitative comparison of effect size.

In short, no meaningful sex-specific patterns were observed. The sex by WhtR interaction term was non-significant (χ^2=0.9; p>0.1) and the effect sizes were comparable across sex (see Supplementary Table S7).

Independent Variable		Female: Estimate/edf	Female: F-/t-score		Male: Estimate/edf	Male: F-/t-score
(Intercept)		1.655	3.600***		7.046	16.835***
Age at Assessment		0.013	4.706***		-0.015	-6.449***
Puberty		0.229	4.679***		0.147	3.354***
Area Deprivation Index		0.008	3.467***		0.009	4.507***
Parental Education (ref. Bachelor's Degree)						
≤ High School Graduate		-0.495	-1.983*		-1.145	-4.804***
High School Graduate		-0.413	-1.910		-1.550	-8.158***
GED or Equivalent		-0.281	-0.971		-1.193	-4.449***
Some College		0.294	1.875		0.108	0.791
Associates Degree Occupational		0.125	0.677		0.044	0.275
Associates Degree Academic		1.347	6.753***		-0.248	-1.389
Master's Degree		0.235	1.991*		-0.311	-2.922**
M.D. or Equivalent		-0.190	-0.916		-0.434	-2.387*
Ph.D. or Equivalent		0.375	1.856		-0.309	-1.810
Household Income		-0.166	-6.252***		-0.283	-11.802***
Self-report Ethnicity (Hispanic)		-0.116	-0.964		-0.004	-0.034
Self-report Race (ref. Black)						
Other/Unknown		1.181	5.962***		0.604	3.298***
White		2.036	15.005***		1.433	11.410***
s(Waist-to-Height Ratio)		1.86	41.76***		2.96	29.5***

Supplementary Table S7. Sex-Stratified Associations Between Internalizing Behaviors and Waist-to-Height Ratio (WHtR). No major sex-differences were observed in the association between internalizing behaviors and WHtR. Summary statistics for the ME-GAM association between internalizing behaviors and WHtR are provided. F-statistics are provided for effect size comparison. Significance codes: * p<0.05, ** p<0.01, *** p<0.001.

There was, however, a significant and positive age x WHtR interaction indicating an increasing strength of association between WHtR and internalizing behaviors across development (see Supplementary Table S8). ANOVA comparison supported the significance of this finding (χ^2=5.7; p=0.016). Note that cross-sectional performance is provided in Supplementary Figures S2 and S3 above.

Independent Variable	Estimate	95% CI	t-value(df)
(Intercept)	5.181	[2.55 7.8]	3.855
Age at Assessment	-0.023	[-0.04 -0.01]	-2.506
Biological Sex at Birth (Male)	-0.157	[-0.36 0.04]	-1.535
Waist-to-Height Ratio	-1.173	[-6.26 3.92]	-0.452
Puberty	0.213	[0.14 0.28]	5.878
Area Deprivation Index	0.009	[0.00 0.01]	2.563
Parental Education (ref. Bachelor's Degree)			
≤ High School Graduate	-0.846	[-1.4 -0.26]	-2.823
High School Graduate	-1.059	[-1.55 -0.57]	-4.233
GED or Equivalent	-0.808	[-1.49 -0.13]	-2.338
Some College	0.130	[-0.23 0.49]	0.700
Associates Degree Occupational	0.058	[-0.37 0.49]	0.267
Associates Degree Academic	0.377	[-0.09 0.85]	1.579
Master's Degree	-0.056	[-0.34 0.23]	-0.382
M.D. or Equivalent	-0.333	[-0.83 0.16]	-1.318
Ph.D. or Equivalent	-0.069	[-0.54 0.40]	-0.289
Household Income	-0.228	[-0.28 -0.17]	-8.180
Self-report Ethnicity (Hispanic)	-0.003	[-0.31 0.30]	-0.019
Self-report Race (ref. Black)			
Other/Unknown	0.883	[0.42 1.35]	3.727
White	1.677	[1.35 2.00]	10.089
Age x Waist-to-Height Ratio	0.044	[0.01 0.08]	2.391

Supplementary Table S8. Age-moderation of the Association Between Internalizing Behaviors and Waist-to-Height Ratio (WHtR). An age by WHtR interaction was observed (ANOVA $p<0.05$, see above) that suggests an increased effect size for the association between WHtR and internalizing behaviors across development. N=31,418 observations across 10,446 unique individuals were considered. The model degree of freedom for parametric terms (df) is 31,394.

Supplementary Materials S5: Full Model Results for Testing the Association Between ABCD-ACEs and Random Slopes (Within-Individual Coupling Between Waist-to-Height Ratio and Internalizing Symptoms)

The fully adjusted model reflecting the association between ABCD-ACEs and the within-individual coupling between WHtR and internalizing symptoms is provided below.

Independent Variable	Estimate	95% CI	t-value(df)
(Intercept)	-2.276	[-3.08 -1.48]	-5.573
ABCD-ACEs	0.639	[0.59 0.69]	26.597
Biological Sex at Birth (Male)	-0.073	[-0.26 0.11]	-0.781
Area Deprivation Index	-0.003	[-0.01 0.00]	0.193
Parental Education (ref. Bachelor's Degree)			
≤ High School Graduate	0.082	[-0.45 0.61]	0.307
High School Graduate	-0.175	[-0.62 0.27]	-0.768
GED or Equivalent	-0.386	[-1.00 0.23]	-1.237
Some College	-0.488	[-0.82 -0.16]	-2.905
Associates Degree Occupational	-0.219	[-0.60 0.17]	-1.119
Associates Degree Academic	-0.514	[-0.93 -0.09]	-2.394
Master's Degree	0.146	[-0.12 0.41]	1.079
M.D. or Equivalent	0.291	[-0.19 0.77]	1.188
Ph.D. or Equivalent	0.238	[-0.21 0.69]	1.030
Household Income	0.080	[0.02 0.14]	2.709
Self-report Ethnicity (Hispanic)	-0.073	[-0.33 0.18]	-0.562
Self-report Race (ref. Black)			
Other/Unknown	0.337	[-0.08 0.76]	1.570
White	0.465	[0.18 0.75]	3.182

Supplementary Table S9. ABCD-ACEs and the Within-individual Coupling Between Internalizing Behaviors and Waist-to-Height Ratio (WHtR). ABCD-ACEs were associated with the within-individual coupling between WHtR and internalizing symptoms after adjustment for potentially confounding factors. N=31,418 observations across 10,446 unique individuals were considered. The model degree of freedom for parametric terms (df) is 31,395.

Supplementary Materials S6: Protective Environments Summary Score

A summary score composed of the four protective environment domains (family, peers, school, community) was derived using an Exploratory Factor Analysis. The one-factor (summary) was specified in R (factanal) using a maximum likelihood estimate and the goodness-of-fit assessed using the chi-square test. A second Confirmatory Factor Analysis (CFA) was conducted (lavaan) to provide additional model fits and indices. CFA fit was modest ($\chi^2(2)=185.6$, $p<10^{-10}$; RMSEA=0.113 [90% CI 0.100–0.128]; CFI=0.778; TLI=0.333; SRMR=0.045).

The summary score explained roughly 13.4% of the total variance with chi-squared statistics ($\chi^2=185.6$, $p<10^{-10}$) suggesting that a single factor is not fully explanatory. This then motivates the inclusion of analyses at the domain-specific level. Factor loadings were comparable across domains ($\beta_{fam}=0.30$, $\beta_{peers}=0.47$, $\beta_{school}=0.34$, $\beta_{comm}=0.33$).

Supplementary Materials S7: Item-Level Moderation of the Association Between ABCD-ACEs and Random Slopes (Within-Individual Coupling Between Waist-to-Height Ratio and Internalizing Symptoms)

Single item analyses consisted of 82 comparisons and therefore our significance threshold was adjusted using a Bonferroni correction (p_{thresh}=0.0006). The model used was a parsimonious linear regression model testing for an interaction between the single-item response and ABCD-ACEs on the within-individual random slopes (Supplementary Figure S5).

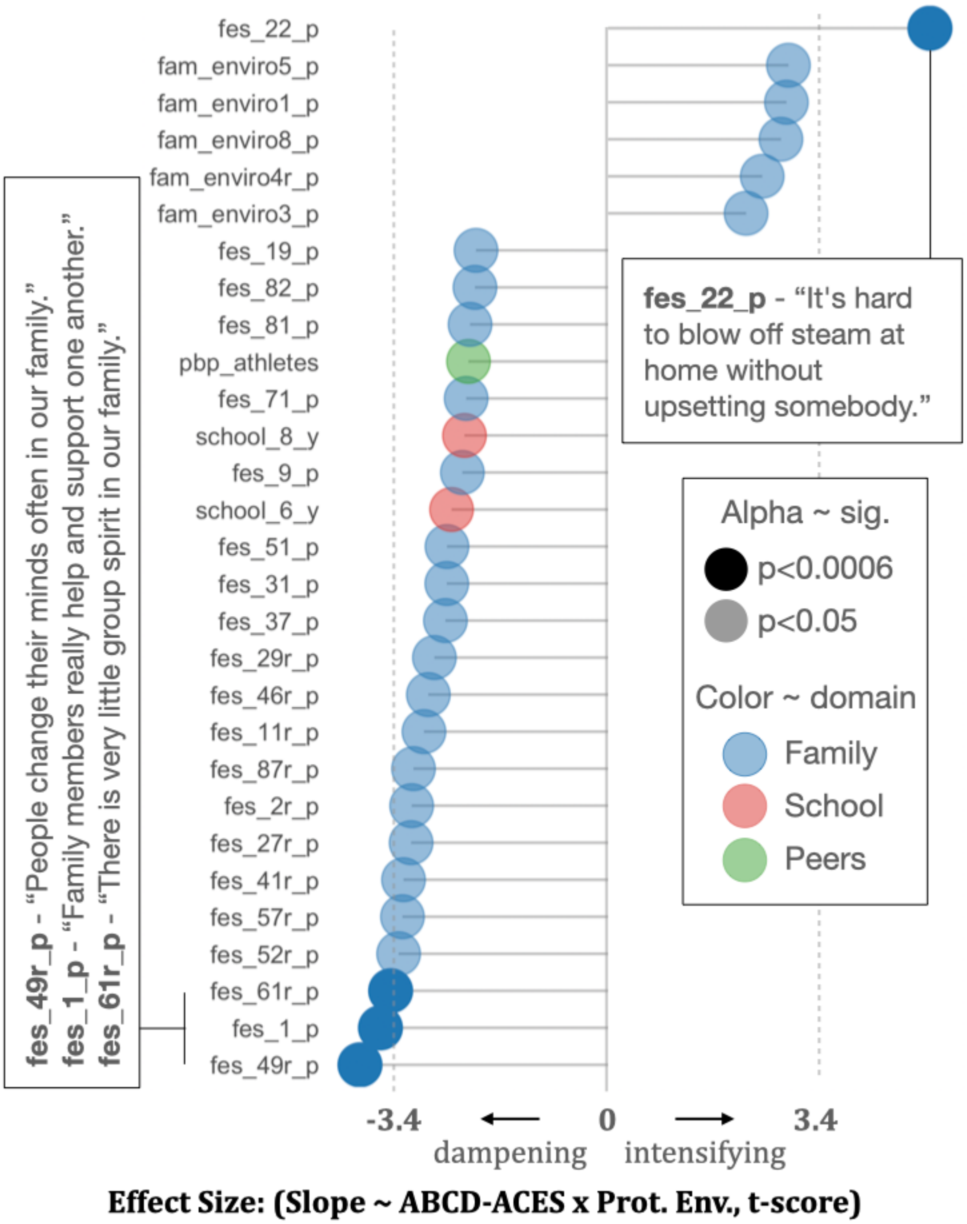

Supplementary Figure S5. Significant Single-item Questions. Four of the family-based protective environment items were significant after multiple comparisons correction (no transparency). Only items significant below p<0.05 are shown (slight transparency). Variable code names are used here for visual clarity, a full description of questions is provided below (Tables S10 and S11).

ABCD Variable	Question Phrasing	t_{inter}	p_{inter}
**fes_22_p**	It's hard to blow off steam at home without upsetting somebody.	5.2	2e-7
**fes_61r_p**	There is very little group spirit in our family (reverse).	-4.0	6.8e-5
**fes_1_p**	Family members really help and support one another.	-3.7	0.003
**fes_49r_p**	People change their minds often in our family (reverse).	-3.5	0.005

Supplementary Table S10. Significant Single-item Questions. In consideration of reverse scoring and direction of effect, items collectively support family sensitivity and consistency as dampeners of the effect of early life adversity on the coupling between waist-to-height ratio and internalizing behaviors.

Domain	Item Name	Item Content	Response Code
Family	fam_enviro1_p	We fight a lot in our family.	1 = True; 0 = False
Family	fam_enviro2r_p	Family members rarely become openly angry.	0 = True; 1 = False
Family	fam_enviro3_p	Family members sometimes get so angry they throw things.	1 = True; 0 = False
Family	fam_enviro4r_p	Family members hardly ever lose their tempers.	0 = True; 1 = False
Family	fam_enviro5_p	Family members often criticize each other.	1 = True; 0 = False
Family	fam_enviro6_p	Family members sometimes hit each other.	1 = True; 0 = False
Family	fam_enviro7r_p	If there is a disagreement in our family, we try hard to smooth things over and keep the peace.	0 = True; 1 = False
Family	fam_enviro8_p	Family members often try to one-up or outdo each other.	1 = True; 0 = False
Family	fam_enviro9r_p	In our family, we believe you don't ever get anywhere by raising your voice.	0 = True; 1 = False
Family	fes_16r_p	We rarely go to lectures, plays, or concerts.	0 = True; 1 = False
Family	fes_17_p	Friends often come over for dinner or to visit.	1 = True; 0 = False
Family	fes_19_p	We are generally very neat and orderly.	1 = True; 0 = False
Family	fes_21_p	We put a lot of energy into what we do at home.	1 = True; 0 = False
Family	fes_22_p	It's hard to blow off steam at home without upsetting somebody.	1 = True; 0 = False
Family	fes_26_p	Learning about new and different things is very important in our family.	1 = True; 0 = False
Family	fes_27r_p	Nobody in our family is active in sports, Little League, bowling, etc.	0 = True; 1 = False
Family	fes_29r_p	It's often hard to find things when we need them in our household.	0 = True; 1 = False
Family	fes_31_p	There is a feeling of togetherness in our family.	1 = True; 0 = False
Family	fes_32_p	We tell each other about our personal problems.	1 = True; 0 = False
Family	fes_36r_p	We are not that interested in cultural activities.	0 = True; 1 = False
Family	fes_37_p	We often go to the movies, sports events, camping, etc.	1 = True; 0 = False
Family	fes_39_p	Being on time is very important in our family.	1 = True; 0 = False
Family	fes_41r_p	We rarely volunteer when something has to be done at home.	0 = True; 1 = False
Family	fes_42_p	If we feel like doing something on the spur of the moment, we often just pick up and go.	1 = True; 0 = False
Family	fes_46r_p	We rarely have intellectual discussions.	0 = True; 1 = False
Family	fes_47_p	Everyone in our family has a hobby or two.	1 = True; 0 = False
Family	fes_49r_p	People change their minds often in our family.	0 = True; 1 = False
Family	fes_51_p	Family members really back each other up.	1 = True; 0 = False

Family	fes_52r_p	Someone usually gets upset if you complain in our family.	0 = True; 1 = False
Family	fes_1_p	Family members really help and support one another.	1 = True; 0 = False
Family	fes_56_p	Someone in our family plays a musical instrument.	1 = True; 0 = False
Family	fes_57r_p	Family members are not very involved in recreational activities outside work or school.	0 = True; 1 = False
Family	fes_59_p	Family members make sure their rooms are neat.	1 = True; 0 = False
Family	fes_61r_p	There is very little group spirit in our family.	0 = True; 1 = False
Family	fes_62_p	Money and paying bills is openly talked about in our family.	1 = True; 0 = False
Family	fes_66_p	Family members often go to the library.	1 = True; 0 = False
Family	fes_67_p	Family members sometimes attend courses or take lessons for some hobby or interest (outside of school).	1 = True; 0 = False
Family	fes_69_p	Each person's duties are clearly defined in our family.	1 = True; 0 = False
Family	fes_71_p	We really get along well with each other.	1 = True; 0 = False
Family	fes_72r_p	We are usually careful about what we say to each other.	0 = True; 1 = False
Family	fes_2r_p	Family members often keep their feelings to themselves.	0 = True; 1 = False
Family	fes_76r_p	Watching TV is more important than reading in our family.	0 = True; 1 = False
Family	fes_77_p	Family members go out a lot.	1 = True; 0 = False
Family	fes_79r_p	Money is not handled very carefully in our family.	0 = True; 1 = False
Family	fes_81_p	There is plenty of time and attention for everyone in our family.	1 = True; 0 = False
Family	fes_82_p	There are a lot of spontaneous discussions in our family.	1 = True; 0 = False
Family	fes_86_p	Family members really like music, art, and literature.	1 = True; 0 = False
Family	fes_87r_p	Our main form of entertainment is watching TV or listening to the radio.	0 = True; 1 = False
Family	fes_89_p	Dishes are usually done immediately after eating.	1 = True; 0 = False
Family	fes_6_p	We often talk about political and social problems.	1 = True; 0 = False
Family	fes_7r_p	We spend most weekends and evenings at home.	0 = True; 1 = False
Family	fes_9_p	Activities in our family are pretty carefully planned.	1 = True; 0 = False
Family	fes_11r_p	We often seem to be killing time at home.	0 = True; 1 = False
Family	fes_12_p	We say anything we want to around home.	1 = True; 0 = False
Peers	pbp_athletes	Are athletes.	1 = None; 2 = A few; 3 = Half; 4 = Most; 5 = All; 999 = Don't know
Peers	pbp_skip_school	Have skipped school.	1 = None; 2 = A few; 3 = Half; 4 = Most; 5 = All; 999 = Don't know
Peers	pbp_church	Go to church once a month or more often.	1 = None; 2 = A few; 3 = Half; 4 = Most; 5 = All; 999 = Don't know
Peers	pbp_suspended	Have been suspended from school.	1 = None; 2 = A few; 3 = Half; 4 = Most; 5 = All; 999 = Don't know
Peers	pbp_good_student	Are excellent students (GPA 3.5 [B+] or higher).	1 = None; 2 = A few; 3 = Half; 4 = Most; 5 = All; 999 = Don't know
Peers	pbp_shoplifted	Have shoplifted occasionally.	1 = None; 2 = A few; 3 = Half; 4 = Most; 5 = All; 999 = Don't know
School	school_2_y	In my school, students have lots of chances to help decide things like class activities and rules.	1 = NO!; 2 = no; 3 = yes; 4 = YES!
School	school_3_y	I get along with my teachers.	1 = NO!; 2 = no; 3 = yes; 4 = YES!
School	school_4_y	My teacher(s) notice when I am doing a good job and let me know about it.	1 = NO!; 2 = no; 3 = yes; 4 = YES!
School	school_5_y	There are lots of chances for students in my school to get involved in sports, clubs, or other school activities outside of class.	1 = NO!; 2 = no; 3 = yes; 4 = YES!

School	school_6_y	I feel safe at my school.	1 = NO!; 2 = no; 3 = yes; 4 = YES!
School	school_7_y	The school lets my parents know when I have done something well.	1 = NO!; 2 = no; 3 = yes; 4 = YES!
School	school_8_y	I like school because I do well in class.	1 = NO!; 2 = no; 3 = yes; 4 = YES!
School	school_9_y	I feel I'm just as smart as other kids my age.	1 = NO!; 2 = no; 3 = yes; 4 = YES!
School	school_10_y	There are lots of chances to be part of class discussions or activities.	1 = NO!; 2 = no; 3 = yes; 4 = YES!
School	school_12_y	In general, I like school a lot.	1 = NO!; 2 = no; 3 = yes; 4 = YES!
School	school_15_y	Usually, school bores me.	1 = NO!; 2 = no; 3 = yes; 4 = YES!
School	school_17_y	Getting good grades is not so important to me.	1 = NO!; 2 = no; 3 = yes; 4 = YES!
Community	comc_phenx_close_knit_p	This is a close-knit neighborhood.	1 = Strongly Disagree; 2 = Disagree; 3 = Neither Agree Nor Disagree; 4 = Agree; 5 = Strongly Agree; 999 = Don't Know; 777 = Refused
Community	comc_phenx_help_p	People around here are willing to help their neighbors.	1 = Strongly Disagree; 2 = Disagree; 3 = Neither Agree Nor Disagree; 4 = Agree; 5 = Strongly Agree; 999 = Don't Know; 777 = Refused
Community	comc_phenx_get_along_p	People in this neighborhood generally don't get along with each other.	1 = Strongly Disagree; 2 = Disagree; 3 = Neither Agree Nor Disagree; 4 = Agree; 5 = Strongly Agree; 999 = Don't Know; 777 = Refused
Community	comc_phenx_share_values_p	People in this neighborhood do not share the same values.	1 = Strongly Disagree; 2 = Disagree; 3 = Neither Agree Nor Disagree; 4 = Agree; 5 = Strongly Agree; 999 = Don't Know; 777 = Refused
Community	comc_phenx_trusted_p	People in this neighborhood can be trusted.	1 = Strongly Disagree; 2 = Disagree; 3 = Neither Agree Nor Disagree; 4 = Agree; 5 = Strongly Agree; 999 = Don't Know; 777 = Refused
Community	comc_phenx_skip_p	If a group of neighborhood children were skipping school and hanging out on a street corner, how likely is it that your neighbors would do something about it?	1 = Very Unlikely; 2 = Unlikely; 3 = Neither Likely Nor Unlikely; 4 = Likely; 5 = Very Likely; 999 = Don't Know; 777 = Refused
Community	comc_phenx_graffiti_p	If some children were spray-painting graffiti on a local building, how likely is it that your neighbors would do something about it?	1 = Very Unlikely; 2 = Unlikely; 3 = Neither Likely Nor Unlikely; 4 = Likely; 5 = Very Likely; 999 = Don't Know; 777 = Refused
Community	comc_phenx_disrespect_p	If a child was showing disrespect to an adult, how likely is it that people in your neighborhood would scold that child?	1 = Very Unlikely; 2 = Unlikely; 3 = Neither Likely Nor Unlikely; 4 = Likely; 5 = Very Likely; 999 = Don't Know; 777 = Refused
Community	comc_phenx_fight_p	If there was a fight in front of your house and someone was being beaten or threatened, how likely is it that your neighbors would break it up?	1 = Very Unlikely; 2 = Unlikely; 3 = Neither Likely Nor Unlikely; 4 = Likely; 5 = Very Likely; 999 = Don't Know; 777 = Refused
Community	comc_phenx_budget_p	Suppose that because of budget cuts the fire station closest to your home was going to be closed down by the city. How likely is it that neighborhood residents would organize to try to do something to keep the fire station open?	1 = Very Unlikely; 2 = Unlikely; 3 = Neither Likely Nor Unlikely; 4 = Likely; 5 = Very Likely; 999 = Don't Know; 777 = Refused

Supplementary Table S11. All Single-item Questions. All single-item questions considered for analysis are provided here for reference.

Supplementary Materials S8: Waist-to-height ratio is relatively age and sex invariant when compared to body-mass-index

Descriptive analyses characterizing the relationship between age and sex, and body composition outcomes BMI and WHtR suggested that age and sex explained substantially more variance in BMI (R^2=6.9%) when compared to WHtR (R^2<0.1%). Notably, sex was not associated with WHtR. Collectively, this demonstrates that relative to BMI, WHtR is relatively age and sex invariant across adolescent development in this sample (Supplementary Figure S6).

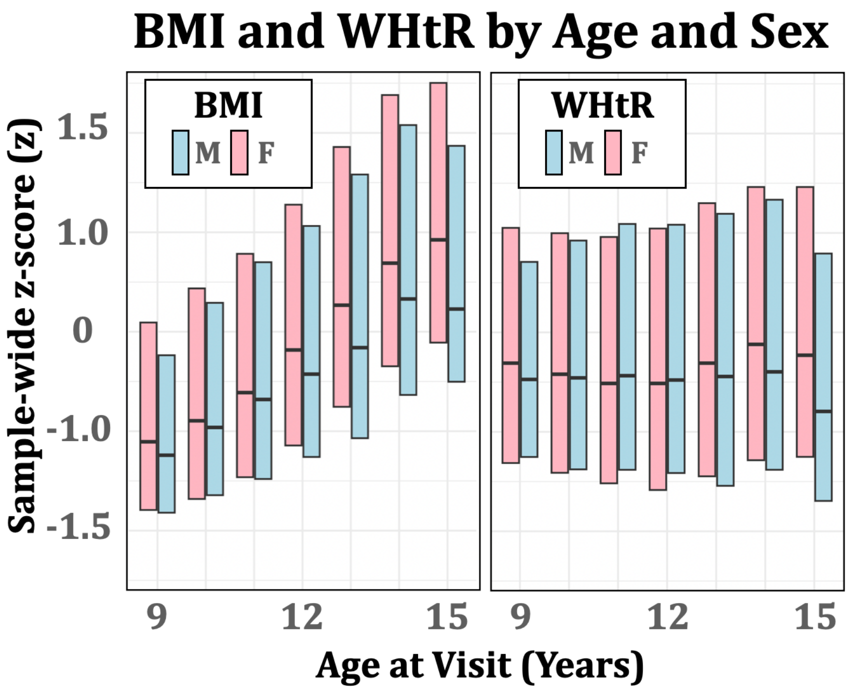

Supplementary Figure S6. Waist-to-Height Ratio (WHtR) Age-Invariance. Relative to BMI, WHtR is not age-dependent, further supporting its use as an index of adiposity across late childhood and adolescence.

Supplementary References

1. Achenbach TM, Edelbrock CS. Behavioral problems and competencies reported by parents of normal and disturbed children aged four through sixteen. Monogr Soc Res Child Dev. 1981;46(1):1-82.
2. Piper BJ, Gray HM, Raber J, Birkett MA. Reliability and validity of Brief Problem Monitor, an abbreviated form of the Child Behavior Checklist. Psychiatry Clin Neurosci. 2014;68(10):759-67.
3. Stinson EA, Sullivan RM, Peteet BJ, Tapert SF, Baker FC, Breslin FJ, et al. Longitudinal Impact of Childhood Adversity on Early Adolescent Mental Health During the COVID-19 Pandemic in the ABCD Study Cohort: Does Race or Ethnicity Moderate Findings? Biol Psychiatry Glob Open Sci. 2021;1(4):324-35.
4. Gonzalez R, Thompson EL, Sanchez M, Morris A, Gonzalez MR, Feldstein Ewing SW, et al. An update on the assessment of culture and environment in the ABCD Study(R): Emerging literature and protocol updates over three measurement waves. Dev Cogn Neurosci. 2021;52:101021.
5. Dennis E, Manza P, Volkow ND. Socioeconomic status, BMI, and brain development in children. Transl Psychiatry. 2022;12(1):33.

STROBE Statement—Checklist of items that should be included in reports of ***cohort studies***

	Item No	Recommendation	Page No
**Title and abstract**	1	(*a*) Indicate the study's design with a commonly used term in the title or the abstract<br>(*b*) Provide in the abstract an informative and balanced summary of what was done and what was found	1-3
**Introduction**			
Background/rationale	2	Explain the scientific background and rationale for the investigation being reported	4-5
Objectives	3	State specific objectives, including any prespecified hypotheses	5
**Methods**			
Study design	4	Present key elements of study design early in the paper	5-6
Setting	5	Describe the setting, locations, and relevant dates, including periods of recruitment, exposure, follow-up, and data collection	6
Participants	6	(*a*) Give the eligibility criteria, and the sources and methods of selection of participants. Describe methods of follow-up<br>(*b*) For matched studies, give matching criteria and number of exposed and unexposed	6
Variables	7	Clearly define all outcomes, exposures, predictors, potential confounders, and effect modifiers. Give diagnostic criteria, if applicable	6
Data sources/ measurement	8*	For each variable of interest, give sources of data and details of methods of assessment (measurement). Describe comparability of assessment methods if there is more than one group	6
Bias	9	Describe any efforts to address potential sources of bias	6-8
Study size	10	Explain how the study size was arrived at	6
Quantitative variables	11	Explain how quantitative variables were handled in the analyses. If applicable, describe which groupings were chosen and why	6-8
Statistical methods	12	(*a*) Describe all statistical methods, including those used to control for confounding<br>(*b*) Describe any methods used to examine subgroups and interactions<br>(*c*) Explain how missing data were addressed<br>(*d*) If applicable, explain how loss to follow-up was addressed<br>(*e*) Describe any sensitivity analyses	6-8
**Results**			
Participants	13*	(a) Report numbers of individuals at each stage of study—eg numbers potentially eligible, examined for eligibility, confirmed eligible, included in the study, completing follow-up, and analysed<br>(b) Give reasons for non-participation at each stage<br>(c) Consider use of a flow diagram	6<br>Fig 1
Descriptive data	14*	(a) Give characteristics of study participants (eg demographic, clinical, social) and information on exposures and potential confounders<br>(b) Indicate number of participants with missing data for each variable of interest<br>(c) Summarise follow-up time (eg, average and total amount)	6<br>Table 1
Outcome data	15*	Report numbers of outcome events or summary measures over time	6-8

Main results	16	(*a*) Give unadjusted estimates and, if applicable, confounder-adjusted estimates and their precision (eg, 95% confidence interval). Make clear which confounders were adjusted for and why they were included<br>(*b*) Report category boundaries when continuous variables were categorized<br>(*c*) If relevant, consider translating estimates of relative risk into absolute risk for a meaningful time period	8-10
Other analyses	17	Report other analyses done—eg analyses of subgroups and interactions, and sensitivity analyses	8-10
**Discussion**			
Key results	18	Summarise key results with reference to study objectives	10
Limitations	19	Discuss limitations of the study, taking into account sources of potential bias or imprecision. Discuss both direction and magnitude of any potential bias	11-12
Interpretation	20	Give a cautious overall interpretation of results considering objectives, limitations, multiplicity of analyses, results from similar studies, and other relevant evidence	10-12
Generalisability	21	Discuss the generalisability (external validity) of the study results	11-12
**Other information**			
Funding	22	Give the source of funding and the role of the funders for the present study and, if applicable, for the original study on which the present article is based	18

*Give information separately for exposed and unexposed groups.

Note: An Explanation and Elaboration article discusses each checklist item and gives methodological background and published examples of transparent reporting. The STROBE checklist is best used in conjunction with this article (freely available on the Web sites of PLoS Medicine at http://www.plosmedicine.org/, Annals of Internal Medicine at http://www.annals.org/, and Epidemiology at http://www.epidem.com/). Information on the STROBE Initiative is available at http://www.strobe-statement.org.
