# Supplementary material for: Early life environment moderates association of body composition and internalizing problems in adolescence
Source: Commun Psychol. 2025 Nov 20;3:163. doi: 10.1038/s44271-025-00336-0 (PMC12634448; doi:10.1038/s44271-025-00336-0)
Supplement: Supplementary file 3 — Reporting Summary [file 44271_2025_336_MOESM3_ESM.pdf]

nature portfolio

Corresponding author(s): Jerod Rasmussen

Last updated by author(s): Jul 15, 2025

Reporting Summary

Nature Portfolio wishes to improve the reproducibility of the work that we publish. This form provides structure for consistency and transparency in reporting. For further information on Nature Portfolio policies, see our Editorial Policies and the Editorial Policy Checklist.

Statistics

For all statistical analyses, confirm that the following items are present in the figure legend, table legend, main text, or Methods section.

n/a	Confirmed	
☐	☒	The exact sample size (*n*) for each experimental group/condition, given as a discrete number and unit of measurement
☐	☒	A statement on whether measurements were taken from distinct samples or whether the same sample was measured repeatedly
☐	☒	The statistical test(s) used AND whether they are one- or two-sided<br>*Only common tests should be described solely by name; describe more complex techniques in the Methods section.*
☐	☒	A description of all covariates tested
☐	☒	A description of any assumptions or corrections, such as tests of normality and adjustment for multiple comparisons
☐	☒	A full description of the statistical parameters including central tendency (e.g. means) or other basic estimates (e.g. regression coefficient) AND variation (e.g. standard deviation) or associated estimates of uncertainty (e.g. confidence intervals)
☐	☒	For null hypothesis testing, the test statistic (e.g. *F*, *t*, *r*) with confidence intervals, effect sizes, degrees of freedom and *P* value noted<br>*Give P values as exact values whenever suitable.*
☒	☐	For Bayesian analysis, information on the choice of priors and Markov chain Monte Carlo settings
☒	☐	For hierarchical and complex designs, identification of the appropriate level for tests and full reporting of outcomes
☐	☒	Estimates of effect sizes (e.g. Cohen's *d*, Pearson's *r*), indicating how they were calculated

Our web collection on statistics for biologists contains articles on many of the points above.

Software and code

Policy information about availability of computer code

Data collection	As a secondary analysis study, no software was used for collection.
Data analysis	All analyses were conducted using R (version 4.3.3). The following R packages were used: ggplot2 for data visualization, mgcv for fitting Generalized Additive Models (GAMs), readr for importing CSV data, tidyr for data reshaping and cleaning, dplyr for data manipulation, car for regression diagnostics, caret for cross-validation. Custom code was written to implement model fitting (linear and non-linear GAMs), compare models using AIC/BIC, perform diagnostics, and generate visualizations. All scripts were executed in RStudio (version 1.3.959) on macOS.

For manuscripts utilizing custom algorithms or software that are central to the research but not yet described in published literature, software must be made available to editors and reviewers. We strongly encourage code deposition in a community repository (e.g. GitHub). See the Nature Portfolio guidelines for submitting code & software for further information.

Data

Policy information about availability of data

All manuscripts must include a data availability statement. This statement should provide the following information, where applicable:

- Accession codes, unique identifiers, or web links for publicly available datasets
- A description of any restrictions on data availability
- For clinical datasets or third party data, please ensure that the statement adheres to our policy

Data Sharing Underlying individual participant data and corresponding data dictionaries are shared as part of the ABCD data repository. All variables used in this

manuscript are denoted by their data dictionary name. All derived data (e.g., random slopes, or "coupling") will be made available upon publication at the NIH Brain Development Cohorts (NBDC) Data Sharing Platform. As with all ABCD data, necessary Data Use Agreements will be required for data download. All code used to produce the manuscripts findings will be made available on JMR's github site (https://github.com/jerodras).

Research involving human participants, their data, or biological material

Policy information about studies with human participants or human data. See also policy information about sex, gender (identity/presentation), and sexual orientation and race, ethnicity and racism.

Reporting on sex and gender	In this study, we use the term 'sex' to refer to a biological attribute included as a key variable in the analyses.<br>Consideration in Study Design and Analysis: Sex was incorporated into the study design as a primary covariate in all main statistical models. Further, we explicitly tested for sex-specific effects in supplementary analyses running models separately for males and females and testing for interactions between key predictors and sex. No interaction effects of note were observed.<br>Method of Determination: The analysis relies on the demo_sex_v2 variable from the ABCD Study dataset, which was treated as a binary variable.<br>Applicability of Findings: The study's primary findings are reported for the entire cohort while adjusting for sex. The conclusion that the waist-to-height ratio (WHtR) is a robust and "largely age- and sex-invariant" predictor of internalizing problems suggests the core findings apply across sexes.
Reporting on race, ethnicity, or other socially relevant groupings	In our manuscript, we included several variables to account for the complex demographic and socioeconomic context of our sample.<br>Socially Constructed Variables Used: The primary socially constructed variables used were race, ethnicity, parental education, household income, and the Area Deprivation Index (ADI). These variables were included in our statistical models as covariates. The purpose of including them was to control for their potential confounding influence on the relationship between body composition, early life adversity, and internalizing problems.<br>Definitions and Classification Methods: This study is a secondary analysis of the Adolescent Brain Cognitive Development (ABCD) Study dataset based primarily on self-report data:<br>Race: The original, more detailed race variables from the ABCD dataset were simplified into three categories for analysis: "White," "Black," and "Other/Unknown".<br>Ethnicity: This was operationalized as a binary variable, categorizing participants as "hispanic" or "nonhispanic".<br>Parental Education and Household Income: To create stable socioeconomic indicators, the maximum reported parental education and household income values were used.<br>Area Deprivation Index (ADI): This is a composite measure of neighborhood-level socioeconomic disadvantage based on residential history data from the ABCD study.<br>Control for Confounding Variables: We controlled for confounding variables by including them simultaneously as covariates in our primary statistical models, including Generalized Additive Mixed Models (GAMM) and Linear Mixed-Effects Models (LMER). By including race, ethnicity, ADI, income, and education as independent predictors in the same model, we ensure that these variables are not used as proxies for one another; rather, the analysis adjusts for the unique variance contributed by each factor. In addition to these covariates, the models also included random intercepts for the study site, family structure, and participant ID (in longitudinal models) to account for non-independence and clustering effects in the data. Finally, sibling discordance analysis and within-person factors (random slopes) added additional control for confounding factors.
Population characteristics	We describe the population in detail as supplementary materials.
Recruitment	This study used data from the Adolescent Brain Cognitive DevelopmentSM (ABCD) Study (https://abcdstudy.org). ABCD is a longitudinal study of ~11,000 participants aged ~10-15 years of age recruited at 21 sites across the United States designed to capture a representative sample of sociodemographic variation from 2017 to present.
Ethics oversight	Secondary analyses on deidentified data is considered non-human subjects research and this designation was filed with, and approved, by the Internal Review Board at the University of California Irvine.

Note that full information on the approval of the study protocol must also be provided in the manuscript.

Field-specific reporting

Please select the one below that is the best fit for your research. If you are not sure, read the appropriate sections before making your selection.

☐ Life sciences ☒ Behavioural & social sciences ☐ Ecological, evolutionary & environmental sciences

For a reference copy of the document with all sections, see nature.com/documents/nr-reporting-summary-flat.pdf

Behavioural & social sciences study design

All studies must disclose on these points even when the disclosure is negative.

Study description	Longitudinal study using Generalized Additive Models and sibling discordance analysis to examine the relationship between body composition and internalizing problems.
Research sample	N=10,446 participants (31,418 observations) ages 10-15 years across five annual visits. Inclusion criteria required complete data for

Research sample	key outcomes (internalizing problems), predictors (waist-to-height ratio [WHtR], body mass index [BMI]), measures of early-life conditions, and covariates.
Sampling strategy	Sampling is intended to be as a normative sample of the US population.
Data collection	Data collection was performed as part of the ABCD study. were sourced from ABCD release 5.1 (Nbaseline=11,868). The following variables were considered: predictors (BMI, WHtR), outcomes (internalizing problems), effect modifiers (ACEs, protective environments), and covariates (age, sex, pubertal status, area deprivation index, parental education, household income, child ethnicity, race). After data cleaning, n=10,446 participants with a total of 31,418 observations remained (see Supplementary Materials S1 for a detailed accounting of data cleaning procedures and Supplementary Table S1 for descriptive measures).
Timing	From 2017 to present.
Data exclusions	Exclusion is specified in Figure 1. Data Inclusion Flow Diagram
Non-participation	Exclusion is specified in Figure 1. Data Inclusion Flow Diagram
Randomization	Participants were not allocated into randomized groups.

Reporting for specific materials, systems and methods

We require information from authors about some types of materials, experimental systems and methods used in many studies. Here, indicate whether each material, system or method listed is relevant to your study. If you are not sure if a list item applies to your research, read the appropriate section before selecting a response.

Materials & experimental systems

n/a	Involved in the study
☒	☐ Antibodies
☒	☐ Eukaryotic cell lines
☒	☐ Palaeontology and archaeology
☒	☐ Animals and other organisms
☒	☐ Clinical data
☒	☐ Dual use research of concern
☒	☐ Plants

Methods

n/a	Involved in the study
☒	☐ ChIP-seq
☒	☐ Flow cytometry
☒	☐ MRI-based neuroimaging

Plants

Seed stocks	*Report on the source of all seed stocks or other plant material used. If applicable, state the seed stock centre and catalogue number. If plant specimens were collected from the field, describe the collection location, date and sampling procedures.*
Novel plant genotypes	*Describe the methods by which all novel plant genotypes were produced. This includes those generated by transgenic approaches, gene editing, chemical/radiation-based mutagenesis and hybridization. For transgenic lines, describe the transformation method, the number of independent lines analyzed and the generation upon which experiments were performed. For gene-edited lines, describe the editor used, the endogenous sequence targeted for editing, the targeting guide RNA sequence (if applicable) and how the editor was applied.*
Authentication	*Describe any authentication procedures for each seed stock used or novel genotype generated. Describe any experiments used to assess the effect of a mutation and, where applicable, how potential secondary effects (e.g. second site T-DNA insertions, mosiacism, off-target gene editing) were examined.*
